# Left Brachiocephalic Vein Stenosis due to the Insertion of a Temporal Right Subclavian Hemodialysis Catheter

**DOI:** 10.1155/2017/9524739

**Published:** 2017-10-22

**Authors:** Eleni I. Skandalou, Fani D. Apostolidou-Kiouti, Ilias D. Minasidis, Ioannis K. Skandalos

**Affiliations:** ^1^Renal Unit “Therapeutiki”, Thessaloniki, Greece; ^2^Surgical Department, General Hospital “Agios Pavlos”, Thessaloniki, Greece

## Abstract

Central vein stenosis/occlusion is a common well-described sequel to the placement of hemodialysis catheters in the central venous system. The precise mechanisms by which central vein stenosis occurs are not well known. Current concepts in central vein stenosis pathophysiology focus on the response to vessel injury model, emphasizing the process of trauma. A case of left brachiocephalic vein stenosis due to the insertion and function of a temporary right subclavian hemodialysis catheter is presented. The purpose of the manuscript is to emphasize that, with the introduction of a temporary subclavian hemodialysis catheter via the right subclavian vein apart from causing concurrent stenosis/infarction of the right subclavian and right brachiocephalic vein, it is also possible to cause stenosis of the left brachiocephalic vein (close to its contribution to the superior vena cava) although the catheter tip is placed in the correct anatomical position in the superior vena cava.

## 1. Introduction

Central vein stenosis/occlusion is a common well-described sequel to the placement of hemodialysis catheters in the central venous system [1, 2]. This situation, in the dialysis patients, is a serious problem and it has a greater impact on the blood inflow compared with stenosis of a peripheral vein, because the central veins represent the final common pathway for blood flow from the periphery to the heart. Central vein stenosis obviates the possibility of creating a new arteriovenous hemodialysis vascular access on the affected side, as the hemodialysis vascular access is frequently lost with the stenosis progression [3]. A consequence of a central vein stenosis is diminished long-term patency of an ipsilateral arteriovenous vascular access. A case of left brachiocephalic vein stenosis due to the insertion and function of a temporary right subclavian hemodialysis catheter is presented.

## 2. Case Presentation

**Figure 1:**
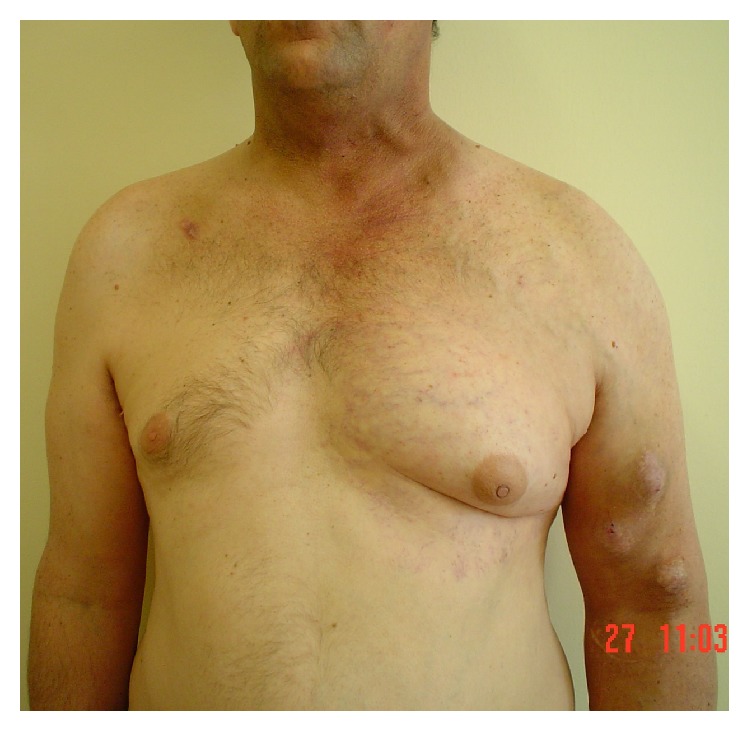
Swelling of the left upper arm and left chest wall. Right subclavian scar.

A 53-year-old man with end stage renal disease, due to renovascular hypertension, started dialysis with right subclavian temporal dialysis catheter four years ago. The introduction of the temporary right subclavian hemodialysis catheter, in the first hospital where the patient was managed, took place with chest X-ray confirmation of the correct catheter tip position in the superior vena cava. A natural radial-cephalic arteriovenous vascular access was created 3 months after that. The performed vascular access needed 6 weeks for its maturation, so the temporal hemodialysis catheter was removed 4.5 months after its insertion. The arteriovenous vascular access was in function for 7 months. The vascular access thrombosis was managed by another ipsilateral proximally brachiocephalic arteriovenous anastomosis which was used directly, due to already maturation of the cephalic vein. In the laboratory investigation, the patient was found to be in procoagulant state with heterozygous factor V (Leiden). A gradually aggravated left upper arm swelling followed by left chest wall swelling and collateral veins dilatation was manifested (Figure 1).

**Figure 2:**
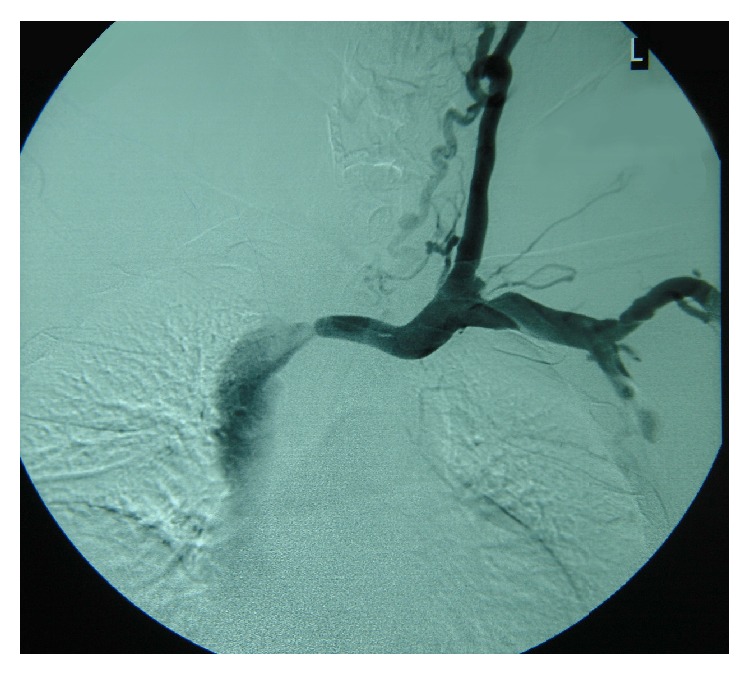
Vascular access angiography: stenosis of the left brachiocephalic vein close to the superior vena cava.

The vascular access angiography showed a large stenosis of the left brachiocephalic vein close to its contribution to the superior vena cava (Figure 2), obviously at the contact point with the tip of the initially introduced temporary right subclavian catheter. Because of the patient's thrombophilic status, a percutaneous angioplasty (PTA) and stenting of the venous stenosis were not attempted.

After that, a right natural radial-cephalic arteriovenous anastomosis was performed and after its maturation the left arm vascular access was ligated. The new vascular access was in use for 16 months, with incidents of venous thrombotic events, progressive right upper arm edema, and venous hypertension (right subclavian vein stenosis). Patient did not show any signs or symptoms related to cerebral oedema and/or brain disorders.

**Figure 3:**
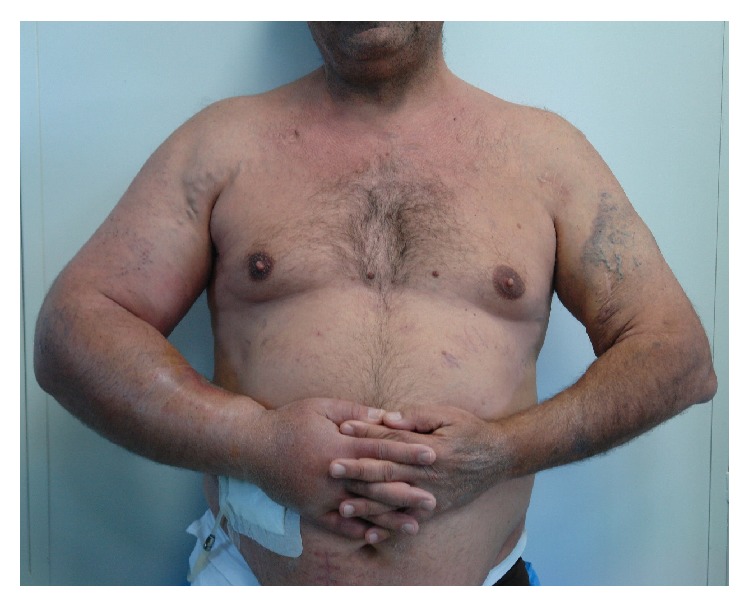
Excessive edema of the right upper arm. Inserted peritoneal dialysis catheter.

We moved on peritoneal dialysis to manage our patient (Figure 3).

## 3. Discussion

The precise mechanisms by which central vein stenosis occurs are not well known. Current concepts in central vein stenosis pathophysiology focus on the response to vessel injury model, emphasizing the process of trauma [4], catheter's material and catheter's contact to venous intima, uremic environment, inflammation, intimal hyperplasia, and a fibrotic response. Regardless of the predominant underlying cause, the final result is the same. There is an upregulation of proinflammatory transcription factors and profibrotic genes, which in turn causes smooth muscle proliferation and thickening of the venous intima [2, 5]. The resulting venous stenosis/obstruction causes venous hypertension. This condition is related to oedema of the arm, difficulty using the vascular access or its loss, and arm dysfunction [2].

The use of silicone catheters for subclavian cannulation is safe and effective to provide temporary vascular access for acute hemodialysis. The incidence of subclavian vein stenosis due to the use of silicone catheters is lower compared to polytetrafluoroethylene and polyurethane catheters. In a comparative study, the polyurethane catheters were also found to be less traumatic than the polytetrafluoroethylene catheters for the venous intima [6].

The initial stenosis is located at the point of the venous wall trauma caused by the insertion of a venous catheter. Furthermore, the direct contact of the catheter with the venous endothelium into the venous lumen and the venous intima trauma caused by the constant venous movement associated with the procedures of breathing and heart function are causes of venous stenosis. The above causes may lead to generation of thrombin, platelet activation, expression of P-selectin, and an inflammatory reaction with increased inflammatory markers [7]. In an autopsy study, there was found that even temporary catheters were associated with focal and local endothelial damage, endothelial denudation and attached organized thrombus, endothelial cells, and collagen [5]. Histological examination of samples of subclavian vein stenosis confirmed endothelial hyperplasia, indicating the presence of fibrous tissue.

Inflammation has an important role in central venous stenosis. The pathology associated with this lesion is characterized by neointimal hyperplasia, which is a common response closely related to vessel injury and inflammation [5, 8]. In our case, the right subclavian catheter tip, lead in a straight route through the right brachiocephalic vein, could be in contact and cause injury to the left brachiocephalic vein endothelium at the point near to its junction to the superior vena cava.

Another mechanism of vascular wall damage is the blood flow turbulence at the catheter's insertion site due to constriction of the vein lumen as well as the turbulence of the blood inflow to the venous blood system from the catheter's tip during the hemodialysis [9]. In our case, the mechanism of intima damage from the blood flow turbulence obviously contributed to the stenosis, due to the proximity or contact of the tip of the right subclavian catheter to the left brachiocephalic vein endothelium at the point near to its junction to the superior vena cava.

Moreover, the hypothesis of uremic environment effect has been supported by recent findings of intimal changes in the cephalic vein of renal failure patients, even prior to arteriovenous fistula (AVF) creation [10]. Intravascular thrombosis can be caused by the release of profibrotic cytokines that are associated with platelet aggregation.

In the 1980s the subclavian venous access was already in a wide use, and a link between subclavian venous catheterization and central venous stenosis was identified back then. In the early 1990s, a removal of the subclavian to the jugular vein catheterization was observed and this option withstood time. Unfortunately, even nowadays, more than 30 years after, the subclavian catheterization continues to be used, even for temporal hemodialysis catheter insertion. Regarding the right subclavian vein catheterization, the venous anatomy should be taken into consideration, as there are two angles in the catheter route: the subclavian to internal jugular junction and the contribution of two brachiocephalic veins to form the superior vena cava. The particularity of this anatomy resulted, in our case, in the left brachiocephalic vein stenosis by a temporal right subclavian hemodialysis catheter. We must note that the initial right subclavian vein catheterization was the cause of all problems that occurred and of such a short time (less than 3 years) of possibility for the patient's therapy by hemodialysis.

## 4. Conclusion

A temporal right subclavian hemodialysis catheter can cause stenosis, not only to the ipsilateral, but also to the heterolateral brachiocephalic vein, with devastating consequences for the creation and function of a hemodialysis arteriovenous vascular access in both arms, as well as for the arm function.

## References

[1] Krishna V. N., Eason J. B., Allon M. (2016). Central Venous Occlusion in the Hemodialysis Patient. *American Journal of Kidney Diseases*.

[2] Yevzlin A. S. (2008). Hemodialysis catheter-associated central venous stenosis. *Seminars in Dialysis*.

[3] Illig K. A. (2011). Management of central vein stenoses and occlusions: The critical importance of the costoclavicular junction. *Seminars in Vascular Surgery*.

[4] Agarwal A. K. (2009). Central Vein Stenosis: Current Concepts. *Advances in Chronic Kidney Disease*.

[5] Forauer A. R., Theoharis C. (2003). Histologic changes in the human vein wall adjacent to indwelling central venous catheters. *Journal of Vascular and Interventional Radiology*.

[6] Tanabe H., Murayama R., Yabunaka K. (2016). Low-angled peripheral intravenous catheter tip placement decreases phlebitis. *Journal of Vascular Access*.

[7] Palabrica T., Lobb R., Furie B. C. (1992). Leukocyte accumulation promoting fibrin deposition is mediated in vivo by P-selectin on adherent platelets. *Nature*.

[8] Torres A., Hernández D., Suria S. (1993). Subclavian catheter-related infection is a major risk factor for the late development of subclavian vein stenosis. *Nephrology Dialysis Transplantation *.

[9] Unnikrishnan S., Huynh T. N., Brott B. C. (2005). Turbulent flow evaluation of the venous needle during hemodialysis. *Journal of Biomechanical Engineering*.

[10] Wali M. A., Eid R. A., Dewan M., Al-Homrany M. A. (2003). Intimal Changes in the Cephalic Vein of Renal Failure Patients before Arterio-Venous Fistula (AVF) Construction. *Journal of Smooth Muscle Research*.

